# Encephalitis caused by *Listeria monocytogenes* in a healthy adult male in China: A case report

**DOI:** 10.1097/MD.0000000000016145

**Published:** 2019-06-21

**Authors:** Na Li, Hua qiong Huang, Gen sheng Zhang, Wen Hua, Hua hao Shen

**Affiliations:** Department of Respiratory and Critical Care Medicine, Second Affiliated Hospital of Zhejiang University School of Medicine, Hangzhou, Zhejiang, China.

**Keywords:** China, encephalitis, immunocompetent, listeria monocytogenes

## Abstract

**Rationale:**

*Listeria monocytogenes* rarely affects immunocompetent adults, and only a few cases of encephalitis caused by *L monocytogenes* in humans have been reported in China.

**Patient concerns:**

: A 37-year-old male patient presented with headache and fever of 38°C to 39°C for 2 days and dysphoria and dystrophy for 1 day.

**Diagnosis:**

The patient was diagnosed as having encephalitis, and his cerebrospinal fluid (CSF) and blood cultures tested positive for *L monocytogenes*.

**Interventions:**

The patient was treated with intravenous vancomycin, meropenem, mannitol, methylprednisolone, and enteral nutrition. The computed tomography (CT) scan showed swelling of the brain and hydrocephalus. The patient was treated with emergent surgery, a ventricular drainage tube was inserted, and the CSF was drained daily.

**Outcomes:**

Despite adequate therapy, the illness was severe and progressed rapidly. The patient died 2 weeks after admission.

**Lessons:**

We report a rare case of *L monocytogenes* encephalitis in a previously healthy immunocompetent adult in China. The patient's CT scans showed increasing brain swelling and hydrocephalus, and the patient's condition progressively deteriorated.

## 1 Introduction

*Listeria monocytogenes* (*L monocytogenes*) is a facultatively anaerobic nonsporulating Gram-positive bacterium.^[1]^ It is known to be responsible for severely invasive disease and can cause sepsis, meningitis, encephalitis, and meningoencephalitis.^[2,3]^ Although listeriosis rarely affects healthy people, high-risk population groups include newborns, elderly individuals, pregnant women, and other immunocompromised patients.^[4]^ Although listeriosis is rare in humans (0.1–10 cases/million), it is associated with a lethality of 30% in cases with neurological involvement.^[5]^ To our knowledge, this is the first reported case of *L monocytogenes* encephalitis in a healthy adult in China.

## 2 Case report

The case report was approved by the Ethics Committee of the Second Affiliated Hospital of Zhejiang University School of Medicine. Informed written consent was obtained from the patient's family for publication of this case report and accompanying images.

A 37-year-old man was admitted to the Emergency Department of our hospital with dysphoria and dystrophy lasting for 1 day. He had suffered from headache and fever of 38°C to 39°C for 2 days before admission. He was otherwise healthy, had no history of drug intake or trauma, and had no previous exposure to chemicals.

**Figure 1:**
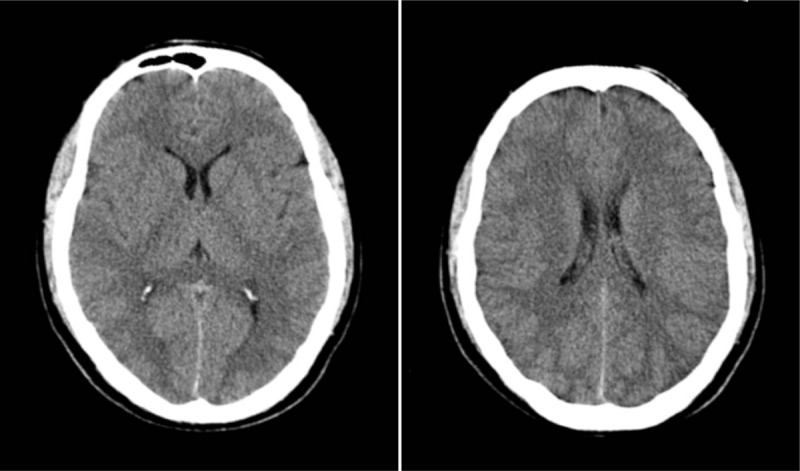
Head computed tomography showing reduction in brain density.

Computed tomography (CT) scan findings were abnormal (Fig. 1). Cerebrospinal fluid (CSF) was obtained via lumbar puncture, and the specimen showed 10 white blood cells (WBCs)/mL, 38% lymphocytes, 128.2 mg/dL protein, 1.8 mmol/L glucose, and 8 U/L adenosine deaminase (ADA). The cerebrospinal pressure was 300 mmH_2_0 (Table 1). Blood and CSF samples were sent to the laboratory for culture. During the examination, the patient lost consciousness and developed tachypnea and coma. He was intubated and sent to the intensive care unit.

**Table 1:**
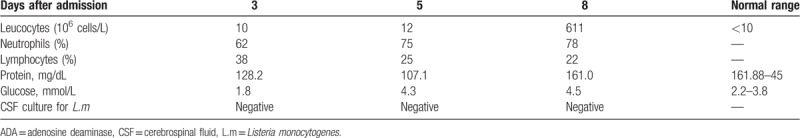
Results of CSF analysis during patient's hospitalization.

On physical examination, he became unconscious (Glasgow Coma Scale score 3/15). His pulse was 115 beats/min, temperature was 38.5°C, blood pressure was 148/78 mmHg, and respiratory rate was 22 breaths/min. His pupils were 4 mm in diameter and nonreactive to light. The patient's neck was stiff, abdomen was soft and non-tender, and no enlargement was noted in the liver and spleen. No abnormal cardiac or pulmonary findings were noted.

The results of the laboratory tests were as follows: WBC count, 18,800 cells/mm^3^; hemoglobin, 12.4 g/dL; mean corpuscular volume, 63.5 fL; platelet count, 67,000 cells/mm^3^; neutrophil count, 18,000 cells/mm^3^; alanine transaminase, 25 U/L; aspartate transaminase, 31 U/L (normal range, 8–35 U/L); C-reactive protein, 301.3 mg/dL; and creatinine, 68 μmol/L. Other findings on tests for antinuclear and anti-DNA antibodies; rheumatoid factors; HBsAg; IgM against HBc; and antibodies against HIV, tuberculosis, cytomegalovirus, and syphilis were all negative. The immunoglobulin (IgG, IgA, and IgM) and T cell (CD3, CD4, CD8, and natural killer cells) levels were all normal. A second lumbar puncture was performed, and this time the CSF sample was found to have 12 WBCs/mL, 25% lymphocytes, 107.1 mg/dL protein, 4.3 mmol/L glucose, and 10 U/L ADA (Table 1).

**Figure 2:**
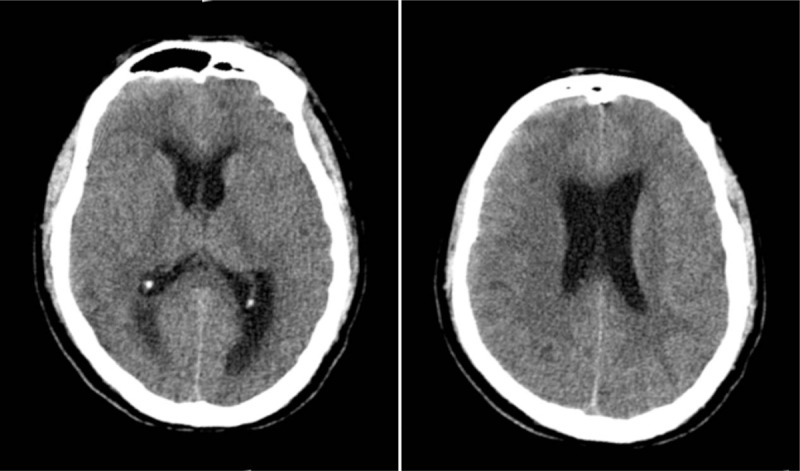
Head computed tomography showing cerebral edema and hydrocephalus.

The patient was diagnosed with encephalitis. As the patient was seriously ill, he was treated empirically with intravenous vancomycin (0.5 g, every 8 hours), meropenem (0.5 g, every 8 hours), mannitol (125 ml, every 8 hours), methylprednisolone (40 mg, once a day), and enteral nutrition. He did not respond to treatment, and the high fever and coma persisted. Two days later, both pupils dilated to 6 mm and were nonreactive to light. A CT scan taken at this time showed swelling of the brain and hydrocephalus (Fig. 2). The patient did not respond to rapid mannitol treatment and was therefore treated with emergent surgery. A ventricular drainage tube was inserted, and the CSF was drained daily.

On the same day, *L monocytogenes* was isolated from blood cultures. *L monocytogenes* is susceptible to linezolid, azithromycin, trimethoprim–sulfamethoxazole, vancomycin, chloromycetin, gentamicin, teicoplanin, moxifloxacin, erythromycin, and rifampicin, and is resistant to penicillin, oxacillin, clindamycin, nitrofurantoin, and cefoxitin. After the positive culture results, rifampicin was administered.

**Figure 3:**
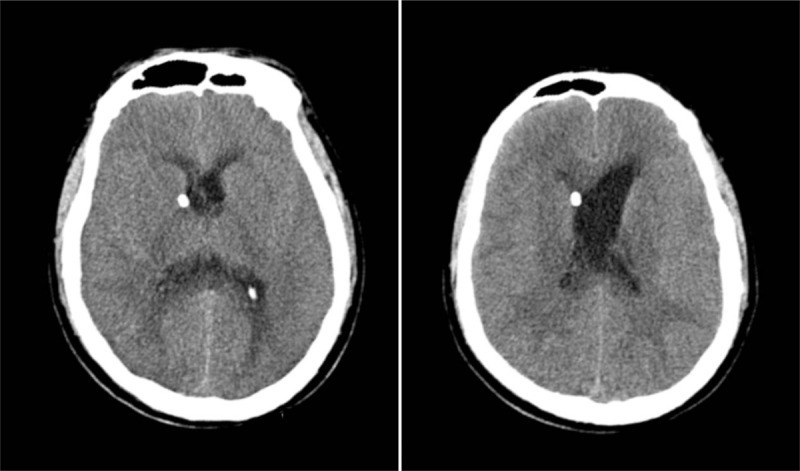
Head computed tomography showing cerebral edema and unshaped sulcus.

Blood culture, cerebrospinal fluid culture (Table 1), and head CT scanning were repeated several times. As the disease continued to progress, the blood culture remained positive for *L monocytogenes* and CT scanning confirmed continued disease progression (Fig. 3). The blood sodium level gradually increased to 172.1 mmol/L, and epilepsy, diabetes insipidus, and shock appeared in succession. The patient died from irreversible hypotension after 2 weeks.

## 3 Discussion

*L monocytogenes* is a pathogenic bacterium in both humans and ruminants. Listeriosis tends to occur after alteration of food habits and administration of immunosuppressive therapies. Although this disease is rare in humans (0.1–10 cases/million), it is the most severe bacterial foodborne infection.^[5]^ Listeriosis can progress to more severe illness, such as sepsis, infection of the central nervous system (CNS), and endocarditis. It has a mortality rate of approximately 30% in cases with neurological involvement, regardless of whether appropriate treatment is administered.^[6,7]^

The incidence of *L monocytogenes* has increased again in recent years, especially in Europe.^[8,9]^ It is unknown why this is occurring, but it may be the result of the relative increase in the size of the population at risk for listeriosis, such as immunosuppressed individuals as well as those with altered food habits and food processing methods.^[3]^

Generally, invasive listeriosis affects immunocompromised individuals and rarely affects healthy individuals. In our case, the patient was previously healthy and had no history of cancer, leukemia, liver cirrhosis, chronic renal failure, organ transplantation, acquired immunodeficiency syndrome, or use of immunosuppressive therapy or corticosteroids. His immunoglobulin (IgG, IgA, IgM) and T cell (CD3, CD4, CD8, and natural killer cells) levels were normal. Therefore, he was considered immunocompetent.

We isolated *L monocytogenes* from the blood culture. It is possible that the patient first became infected with the bacteria via blood-borne transmission, and then the bacteria got into the CNS through the blood–brain barrier. The CSF sample showed 10 WBCs/mL, and CSF culture was negative. A special symptom in this patient was rapid deterioration of consciousness. CT findings became abnormal on day 2, revealing cerebral edema. Based on these data, the patient was diagnosed as having *L mon*ocytogenes encephalitis.

Although the CNS is involved, brain CT scans of listeriosis patients are normal in most cases. In encephalitis caused by *L monocytogenes*, hydrocephalus is a very rare complication. Yang et al reported a case of *L. monocytogenes* meningitis with acute hydrocephalus detected on a brain CT scan. In this patient, the CSF was drained daily. The patient's mental status recovered completely after 4 days, and a good outcome was achieved.^[10]^ In our case, the patient's condition progressively deteriorated. Because *L monocytogenes* can cross the blood–brain barrier and gain access to the CNS, transmission of the infection to the patient's brain was rapid and fatal. In the first few days, the pathogen was not detected, and the patient was empirically treated with intravenous vancomycin and meropenem. Although the bacteria were sensitive to vancomycin, rifampicin was added to the treatment because of the rapid progression of the disease. The patient's CT scans showed increasing brain swelling and hydrocephalus. After receiving antibiotic therapy for 2 weeks, the patient died from severe complications of CNS infection, including diabetes insipidus and hypotension.

*L monocytogenes* meningitis in immunocompetent adults has been reported in the literature, but *L monocytogenes* encephalitis has been rarely reported, and most patients with this illness have good outcomes. In our case, the therapy was unsuccessful. This may be because of 2 important reasons: the CNS was susceptible to *L monocytogenes*, and the empirical treatment administered was inappropriate. As reported previously, using first-line antibiotics such as ceftriaxone or vancomycin for empirical treatment of community-acquired meningitis may have catastrophic results if it is caused by *L monocytogenes*. Cephalosporins are ineffective in treating *Listeria,* and vancomycin is known to have variable results.^[4,11]^ Although it remains unclear which antibiotics are optimal for the treatment of *L monocytogenes* meningitis, ampicillin used concomitantly with a synergistic aminoglycoside is the preferred agent for therapy. Additionally, a study has reported some antimicrobial effects of rifampicin and meropenem against *L monocytogenes*.^[12]^ Owing to the high mortality rate associated with *L monocytogenes*, it should be considered when screening the potential causes of community-acquired bacterial encephalitis in immunocompetent patients. Proper and timely diagnosis as well as antibiotic therapy is vital to ensure good outcomes in patients with *L monocytogenes* encephalitis.

## Acknowledgment

The authors thank the patient's family who agreed to our report with anonymity.

## Author contributions

**Conceptualization:** Hua hao Shen.

**Data curation:** Na Li.

**Investigation:** Na Li, Hua qiong Huang.

**Supervision:** Hua qiong Huang, Gen sheng Zhang.

**Writing – original draft:** Na Li, Wen Hua.

**Writing – review & editing:** Hua hao Shen.
